# Development and validation of a quantitative Orthopoxvirus immunoassay to evaluate and differentiate serological responses to Mpox infection and vaccination

**DOI:** 10.1016/j.ebiom.2025.105622

**Published:** 2025-02-22

**Authors:** Joanne Byrne, Gurvin Saini, Alejandro Garcia-Leon, Dana Alalwan, Peter Doran, Alan Landay, Liem Binh Luong Nguyen, Cathal O'Broin, Stefano Savinelli, Jane A. O'Halloran, Aoife Cotter, Mary Horgan, Christine Kelly, Corinna Sadlier, Eoghan de Barra, Virginie Gautier, Patrick W.G. Mallon, Eoin R. Feeney, All Ireland Infectious Diseases Cohort Study

**Affiliations:** a Centre for Experimental Pathogen Host Research (CEPHR), University College Dublin, Belfield, Dublin 4, Ireland; b Department of Infectious Diseases, St Vincent's University Hospital, Elm Park, Dublin 4, Ireland; c School of Medicine, University College Dublin, Belfield, Dublin 4, Ireland; d Department of Medicine, University of Texas Medical Branch, Galveston, TX, USA; e Université Paris Cité, France; f CIC Cochin Pasteur, Assistance Publique–Hôpitaux de Paris, Paris, France; g Department of Infectious Diseases, Mater Misericordiae University Hospital, Eccles St, Dublin 7, Ireland; h Department of Infectious Diseases, Cork University Hospital, Wilton, Co Cork, Ireland; i Department of Infectious Diseases, Beaumont Hospital, Beaumont, Dublin 9, Ireland; j Department of International Health and Tropical Medicine, Royal College of Surgeons in Ireland, Dublin, Ireland

**Keywords:** Monkeypox virus, Mpox, Orthopoxvirus, MVA-Vaccine, Immunoassay

## Abstract

**Background:**

The Mpox outbreak, caused by Monkeypox virus (MPXV), underscores the need for a serological assay to assess Mpox immunity. Modified Vaccinia Ankara (MVA) vaccine, an attenuated vaccinia virus (VACV), is authorised for Mpox prevention. We aimed to develop a quantitative immunoassay to differentiate infection- and vaccination-induced immunity and explore serological responses to Mpox infection and vaccination.

**Methods:**

We evaluated an electrochemiluminescence assay targeting IgG to 10 MPXV and 3 VACV antigens in plasma from adults in a cohort study with previous Mpox, MVA-vaccination, or historical controls. Sensitivity and specificity to distinguish i) seropositive versus naive and ii) infection- versus vaccination-induced seropositivity were determined using ROC curves. Antibody kinetics were analysed with generalised additive models.

**Findings:**

Eight of the thirteen IgG antibodies showed significant titre differences across groups identifying three key antigens: MPXVB6R, MPXVA27L, and VACVB5. A VACVB5 IgG titre of 0.082 IgG normalised units (nu) offered 74% (95% CI: 59–82%) sensitivity and 81% (73–96%) specificity for previous antigen exposure (infection or vaccine). For infection alone, an MPXVB6R IgG titre of 0.075 IgGnu provided 89% (82–98%) sensitivity and 94% (86–100%) specificity. To differentiate infection from vaccination-induced seropositivity, the sum of MPXVA27L IgG and the B6R/VACVB5 ratio provided 89% (80–96%) sensitivity and 80% (74–84%) specificity. VACVB5 IgG titres declined over time, with higher titres post-Mpox than post-vaccination (*p* < 0.0001).

**Interpretation:**

This assay demonstrates high sensitivity and specificity in quantifying and differentiating between antibody responses to Mpox infection and vaccination. Post-Mpox antibody responses were higher than post-vaccination, though both waned over time.

**Funding:**

10.13039/100010414Health Research Board (MONKEYVAX-2022-1), 10.13039/501100001631University College Dublin School of Medicine.

**Research in context**

**Evidence before this study**

Until 2022, Mpox was considered a neglected tropical disease with limited data on the host immune response to vaccination or infection. Previous studies have largely inferred host immunity to Mpox from smallpox vaccination. While the Modified Vaccinia Ankara (MVA) smallpox vaccine was given emergency authorisation for Mpox prophylaxis, its real-world long-term efficacy remained uncertain. A meta-analysis has estimated the efficacy of two doses of MVA-BN vaccine to prevent Mpox at 81.8% (95% CI: 65.0−89.1%). However, emerging evidence has raised concerns regarding the durability of protection with clusters of cases in vaccinated individuals and reinfections. Furthermore, Orthopoxviruses are large dsDNA viruses with extensive antigenic cross-reactivity which have confounded immunoassay development. Additionally, there is no approved assay to differentiate between infection- and vaccination-induced immunity, highlighting a significant gap in the understanding of humoural responses to Mpox.

**Added value of this study**

This study has significantly advanced the understanding of immune responses to Mpox by developing a quantitative, serologic assay that targets three key OPXV antigens: MPXV B6R, A27L, and VACV B5. This assay is both sensitive and specific to quantify antibody responses to both MVA-vaccination (VACVB5) and infection (MPXV B6R) and can differentiate between post infection and post vaccination immune responses [(B6R/VACB5) + A27L]. Using this assay, we explored the dynamics of antibody responses to Mpox vaccination and infection over time and demonstrated that circulating antibodies post MVA-vaccination are significantly lower than post infection and wane considerably significantly over time.

**Implications of all the available evidence**

Our assay offers high sensitivity and specificity, enabling a precise evaluation of immune responses in individuals exposed to Mpox or vaccinated with MVA. The study addresses an urgent need for a practical, scalable assay that can be implemented in low- and middle-income countries (LMICs) like the DRC, where Mpox cases are rising and existing diagnostic tools are insufficient. Unlike labour-intensive and costly live virus neutralisation assays, this assay can be widely applied in large-scale vaccine trials and routine clinical settings. This is particularly crucial in resource-limited regions, where accurate serological surveillance can assist in controlling outbreaks. Overall, our study provides a valuable tool for global public health efforts to monitor and respond to the ongoing Mpox public health emergency.

## Introduction

Human Mpox infection was first reported in the Democratic Republic of Congo (DRC) in 1972.^1^ Historically, Mpox outbreaks were endemic in West and Central Africa, typically linked to zoonotic transmission. Whilst sporadic cases of Mpox have occurred outside of these areas, an unprecedented outbreak occurred in May 2022, with autochthonous cases of clade IIb MPXV outside of endemic countries, resulting in the WHO declaring Mpox a public health emergency in July 2022.^2^ To date, over 99,000 cases of MPXV have been reported, primarily through sexual transmission among gay and bisexual men who have sex with men (gbMSM).^3^ A more recent 2023 clade I Mpox virus outbreak in the Democratic Republic of Congo and neighbouring countries is ongoing and has resulted in almost 19,000 cases and 1007 deaths.^4^ In contrast to the 2022 outbreak, more than half the cases have occurred in women, female sex workers are overrepresented among affected individuals (29%), and children at particular risk of severe disease.^5^ This led to the WHO declaring Mpox a public health emergency again in August 2024.^6^

Monkeypox virus (MPXV), a member of the Orthopoxvirus (OPXV) genus is the causative agent of human Mpox infection. Other OPXVs include Variola virus, the causative agent of smallpox and Vaccinia virus used in smallpox vaccines. OPXVs are large dsDNA viruses with extensive antigenic cross-reactivity.^7^ Within MPXV, there are two clades with 99.57% homology between clade I and clade II.^8^ In response to the 2022 Mpox outbreak, a third-generation smallpox vaccine, Modified Vaccinia Ankara (MVA), was given emergency authorisation for Mpox pre-exposure and post-exposure prophylaxis, based on its immunogenicity from phase I to III trials,^9^, ^10^, ^11^ and prior evidence of fewer clinical Mpox cases in case contacts with a history of smallpox vaccination,^12^ rather than evidence of real-world clinical efficacy. A meta-analysis has estimated the efficacy of two doses of MVA-BN vaccine to prevent Mpox at 81.8% (95% CI: 65.0−89.1%) and reported a positive correlation between vaccine effectiveness and Vaccinia virus (VACV)-binding antibody titres.^13^ However, there is emerging evidence indicating low levels of MPXV neutralising antibodies, raising concerns about the durability of protection post vaccination.^14^, ^15^, ^16^

Until recently, Mpox has been regarded as a neglected tropical disease and human immunity to Mpox has not been extensively characterised. Most inferences regarding host immunity are derived from studies on other OPXV, such as smallpox, which confers life-long immunity.^17^ Although similar humoural antigen recognition between MPXV-infected and smallpox-vaccinated participants across multiple poxvirus antigens was observed in one study,^18^ significantly higher anti-MPXV IgG in convalescent individuals versus vaccinated individuals has also been reported.^19^

Case numbers of clade IIb Mpox have decreased globally, believed to be secondary to a combination of vaccination-induced and infection-derived immunity and behavioural modification.^20^, ^21^, ^22^ However, case clusters in vaccinated individuals and reports of reinfections have raised concerns about the durability of both vaccine-induced and infection-induced immunity and whether booster vaccine doses are required.^23^

There is no approved serological assay to quantify the humoural immune response to infection and vaccination against Mpox and to discriminate between the antibodies produced following vaccination versus those produced following infection. To address these gaps, we aimed to develop a quantitative immunoassay to multiple OPXV antigens, to explore serological immunity to Mpox and MVA Vaccination over time and define which antibodies or combination of antibodies best differentiate between infection and vaccination induced immunity.

## Methods

### Ethics

The All-Ireland Infectious Diseases (AIID) Cohort Study is a prospective, multi-centre, observational study recruiting individuals attending hospital with issues pertaining to infectious diseases (approved by the National Research Ethics Committee in Ireland, reference 20-NREC-COV-056).

### Study design and participants

Adult (≥18 years) participants provided written, informed consent for collection of clinical data and blood samples for biobanking (stored at −80 °C). Self-reported participant race and ethnicity data was collected. From the AIID Cohort, we selected four groups; participants with a history of PCR-confirmed MPXV Clade IIb disease (Mpox group), those who received MVA vaccine for Mpox prophylaxis (MVA Vaccine group), a group sampled prior to the Mpox outbreak of 2022 and without a history of prior smallpox vaccine, born after the cessation of smallpox vaccine in 1972 in Ireland or 1989 in the Americas, (Control group),^24^^,^^25^ and a group, born before the cessation of smallpox vaccine in 1972 with a history of childhood smallpox vaccination (Childhood Vaccine group). All participants in the MVA Vaccine group received the MVA-BN vaccination (JYNNEOS) as per national immunisation guidelines.^26^

### Selection of MPXV and VACV antigens

MPXV and VACV genome and protein sequences were compared using the clade I MPXV reference genome Zaire-96-I-169 (NCBI:txid619591) and the VACV Copenhagen strain genome (NCBI:txid10249) on the NCBI virus database. Ten MPXV antigens were selected spanning a range of functional viral proteins within the two major forms of infectious virus during the MPXV replication cycle: intracellular mature virus (IMV) and extracellular enveloped virus (EEV). This selection also targeted both MPXV antigens with VACV homologues defined as antigens that target common structural protein domains, and those without, ensuring comprehensive coverage of the antigenic diversity (Supplementary Table S1). Eight of the ten MPXV antigens had homologous VACV antigens corresponding to the MPXV A33R, H3L, A35R, D13L, B6R, A29, M1R, and E8L. Of these, we chose three VACV antigens for inclusion in assay development (VACVB5, VACVA33R, and VACVA27L) that matched MPVX antigens B6R, A35R, and A29 respectively. Additionally, two chosen MPXV antigens were absent from the MVA-vaccination; A26L did not have a corresponding VACV homologue and although A27L has a homologue (VACV A25), it is absent from the modified non-replicating strain used in the MVA Vaccine.^27^

### Electrochemiluminescence assay

Each antigen was reconstituted in sterile water, stored at −80 °C, and used to coat 96-well high-bind plates (MSD, Rockville, MD) at 5 pmol per well overnight at 4 °C. Plates were washed with PBS-Tween (Bio Sciences Ltd., Ireland), blocked with 1% blocker A (MSD) for 120 min at room temperature, and then plasma diluted 1:500 in MSD D100 was added. Biological controls per plate included plasma from Mpox-infected and MVA-vaccinated participants, and a negative control. After a 90-min incubation, plates were washed and MSD SULFO-TAG-labelled anti-human IgG secondary antibody was added and incubated for 1 h. Finally, MSD GOLD read buffer A was added, and plates were analysed using the MESO QuickPlex SQ 120 and MSD Discovery Workbench Software Version 4.0. IgG titres were normalised and expressed as IgG normalised units (IgGnu). This assay is patent pending under the patent application entitled ‘A method of quantifying and differentiating serological responses to Mpox infection and vaccination’, filed on 29th October 2024, UK 2415898.2. Detailed methods are provided in the Supplementary Materials.

### Role of funders

This work was supported by Health Research Board (grant number MONKEYVAX-2022-1). This work was additionally supported by the University College Dublin School of Medicine Translational Seed Fund. The funders had no role in study design, data collection, data analyses, interpretation, or writing of report.

### Statistics

To compare characteristics within groups, continuous variables such as age and time were summarised using median and interquartile range (IQR), while categorical variables were summarised with frequencies and percentages. We compared IgG titres and the difference in MPXV/VACV antigen ratio between groups, using the Kruskal–Wallis test, with post-hoc Dunn's test or Mann–Whitney U test, respectively. To determine sensitivity and specificity of each antigen in quantifying immunity, receiver operating characteristic (ROC) curves, area under the curve and Youden Index were constructed using the *pROC* package in R. In our ROC analyses, we compared known infection and vaccination cases to negative controls, and infection versus vaccination cases. To determine the change in antibody responses over time to vaccination and infection, scatter plots, with superimposed curves fitted using generalised additive mixed models (GAMMs), were used to depict the non-linear relationship between time since vaccination or infection and quantitative antibody levels. These models used a Gaussian function and time since vaccination or infection fitted as a spline. GAMMs were fitted using the *mgcv* package in R, incorporating individual participants as a random effect and an autocorrelation error structure. To compare antibody responses to infection and vaccination, Welch Two Sample t-test was used to compare the aggregated predicted means using these models. Statistical analysis was performed using R software (Version 4.4.1).

## Results

### Study population

A total of 295 participants provided 383 samples for analysis across the four groups; Mpox group 54 samples from 28 participants, MVA vaccine group 229 samples from 167 participants, Control group 78 samples from 78 participants, and Childhood Vaccine group 22 samples from 22 participants. Participant clinical characteristics are listed in Table 1. All participants were assigned male sex at birth, and the age (median [IQR]) of participants was similar across the groups: Mpox (32 [29–39]), MVA Vaccine (35 [30–41]), and Control (32 [29–38]) with the exception of the older Childhood Vaccine group (72 [56–78]). Of the total cohort of 295, 60 (20%) were living with HIV, with the prevalence of HIV similar across groups. The Mpox samples were taken at a median (IQR) of 277 (11–449) days from onset of symptoms. In the MVA Vaccine group, all participants were sampled at a median (IQR) of 238 (138–327) days post completion of vaccination course.

**Table 1:** Demographics of study population.

	Mpox (n = 28)	MVA-vaccine (n = 167)	Control (n = 78)	Childhood vaccine (n = 22)
Samples	54	229	78	22
Age (median (IQR))	32 (29–39)	35 (30–41)	32 (29–38)	72 (56–78)
Male sex at birth (n(%))	28 (100%)	167 (100%)	78 (100%)	22 (100%)
Race (n(%))				
Asian	0 (0%)	12 (7%)	7 (9%)	0 (0%)
Black African	0 (0%)	1 (1%)	0 (0%)	0 (0%)
Caucasian	28 (100%)	154 (92%)	71 (91%)	22 (100%)
Ethnicity: (n(%))				
Hispanic or Latino	11 (39%)	57 (34%)	16 (21%)	1 (5%)
Not Hispanic or Latino	15 (54%)	110 (66%)	62 (79%)	21 (95%)
Middle Eastern	2 (7%)	0 (0%)	0 (0%)	0 (0%)
HIV (n(%))	5 (17.8%)	32 (19.2%)	19 (24.4%)	4 (18.2%)
Childhood smallpox vaccine (n(%))^a^				
Yes	2 (7.1%)	15 (9%)	0 (0%)	22 (100%)
No	21 (75%)	136 (81.4%)	78 (100%)	0 (0%)
Unknown	5 (17.9%)	16 (9.6%)	0 (0%)	0 (0%)
Sample days from infection/vaccination (median (IQR))	277 (11–449)^b^	238 (138–327)^c^	NA	NA

a History of childhood smallpox vaccination was determined based on defined thresholds: Participants born before 1972 were assumed to have received the vaccination. Participants born after 1972 in Ireland were considered unvaccinated, while the vaccination status of participants born in the Americas between 1972 and 1989 was classified as unknown.

b Multiple samples in the same participants in the Mpox group were taken at a median (IQR) 267 (88–407) days apart.

c Multiple samples in the same participants in the MVA Vaccine group were taken at a median (IQR) 91 (84–170) days apart.

### IgG titres across groups

**Fig. 1:**
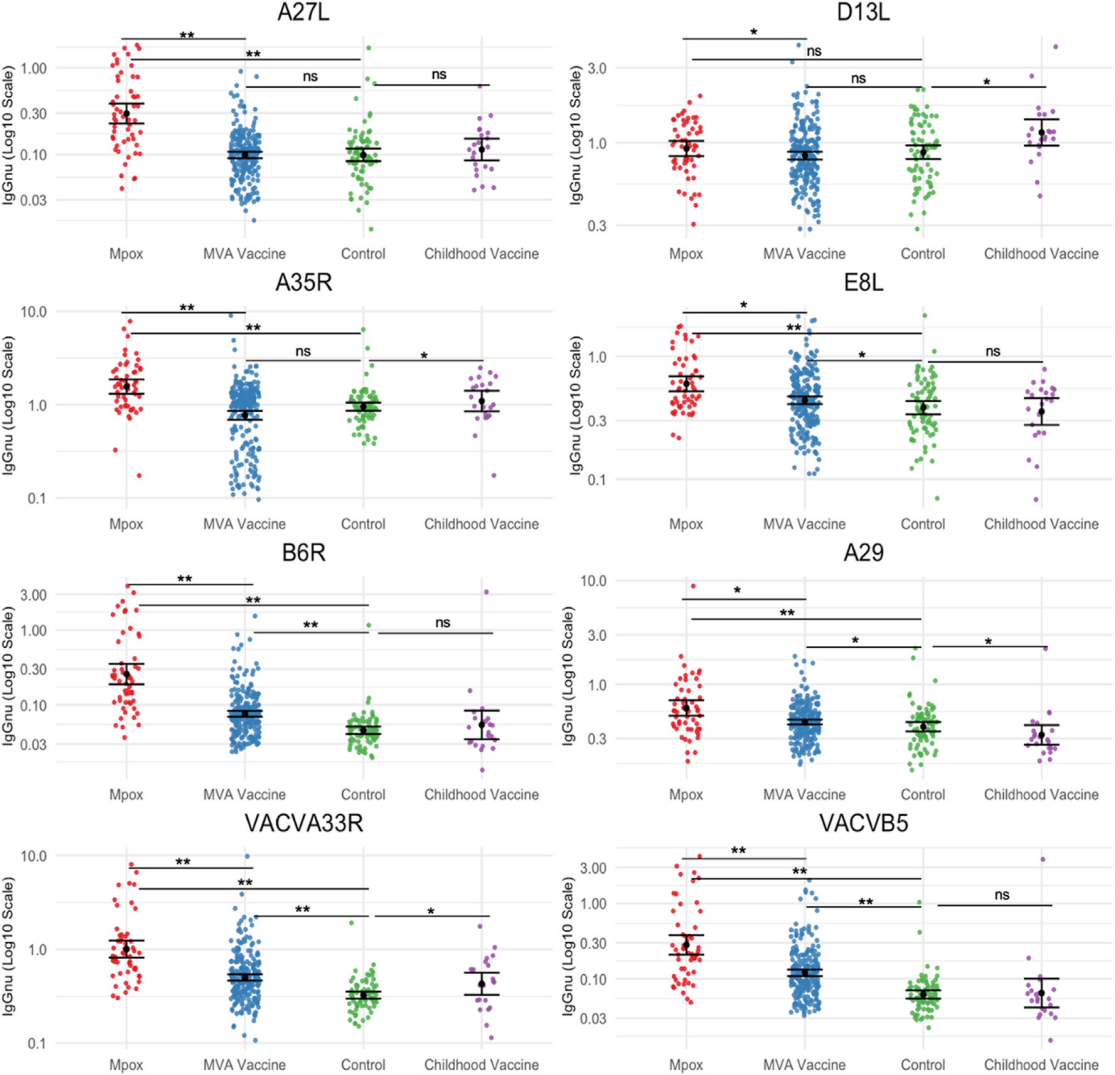
**Significantly different IgG titres across groups**. Each graph represents the IgG titres for that antigen for each group. Mpox (n = 54), MVA Vaccine (n = 229), Control (n = 78), Childhood Vaccine (n = 22). The error bars represent the geometric mean titre (GMT) and the 95% confidence interval of the GMT for each group. Each antigen in this figure had significantly different titres across the four groups by Kruskal–Wallis Test (all *p* < 0.05). Comparisons shown represent *p* values as per post-hoc Dunn's test to assess differences in titres between Mpox, MVA Vaccine, Control and Childhood Vaccine group with ∗*p* = 0.0001–0.05, ∗∗*p* < 0.0001, and ns = not significant.

Eight of the 13 target IgG titres significantly differed when compared across the four groups: the MPVX antigens A27L, D13L, A35R, E8L, B6R, A29, and VACV homologues VACVA33R and VACVB5 (all *p* < 0.02, Fig. 1). The remaining five target antibody titres (A33R, H3L, A26L, M1R, and VACV A27L) did not significantly differ between groups and were not part of further assay development (Supplementary Figure S1). Using the post-hoc Dunn's test, between group differences were between the Mpox group and the Control group in all but one of these eight antigens (D13L) (Supplementary Table S2). When the MVA Vaccine group was compared to the Control group, MPXV antigens E8L, B6R, A29, and VACV antigens B5 and A33R differed significantly (all *p* < 0.03, Supplementary Table S2). When the Childhood Vaccine group was compared to the Control group, MPXV antigens D13L, A35R, A29, and VACVA33R differed significantly (all *p* < 0.05, Supplementary Table S2).

### Relationship between antibodies targeting MPXV and their VACV homologoues

To compare IgG titres of the two MPXV antigens that had matched VACV homologues, we calculated the ratio of IgG titres for the MPXV antigen and the corresponding VACV homologue and compared them between the Mpox and MVA Vaccine groups. The MPXV B6R/VACVB5 ratio was significantly higher in the Mpox group (0.85 [0.79–1.05]) versus the MVA Vaccine group (0.68 [0.57–0.77], *p* < 0.0001). In contrast there was no significant difference in the MPXVA35R/VACVA33R ratio between groups (Supplementary Figure S2).

### Assay performance at determining antibody responses post infection and vaccination

**Fig. 2:**
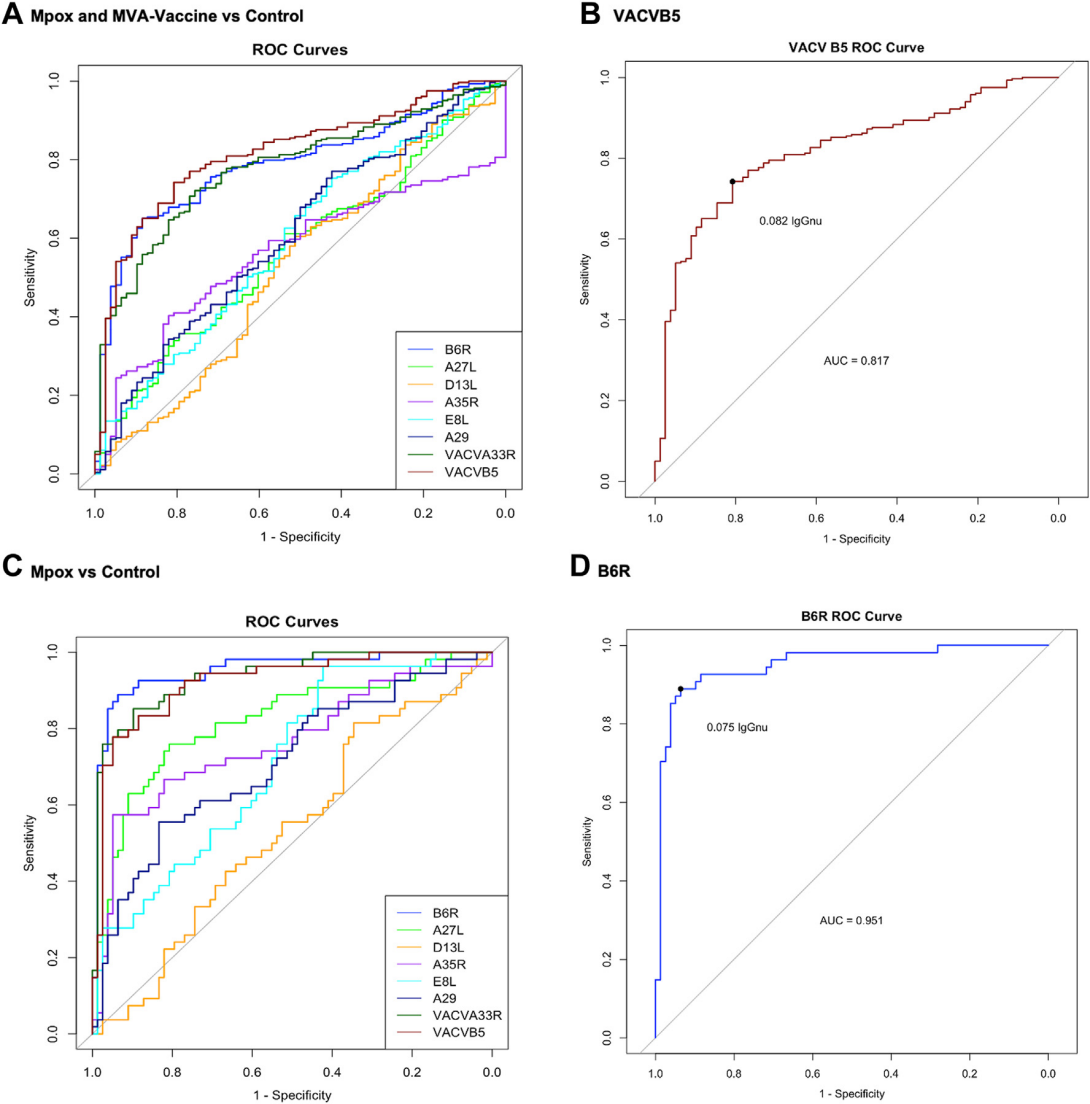
**Receiver operating characteristic (ROC) curves**. **A:** ROC curve for all antigens generated by comparing positive samples (Mpox and MVA Vaccine group, n = 283) to negative samples (Control group, n = 78). **B:** ROC curve for VACVB5 generated by comparing positive samples (Mpox and MVA Vaccine group, n = 283) to negative samples (Control group, n = 78). **C:** ROC curve for all antigens generated by comparing positive samples restricted to infection only (Mpox group, n = 54) to negative samples (Control group, n = 78). **D:** ROC for MPXV B6R generated by comparing positive samples restricted to infection only (Mpox group, n = 54) to negative samples (Control group, n = 78).

To evaluate the performance of the assay to determine antibody responses post infection and vaccination, we constructed ROC curves for each of the eight chosen antigens, using the 283 samples from the Mpox and MVA Vaccine groups as true positives and the 78 Control samples as true negatives respectively. The area under the curves (AUC) ranged from 0.528 for D13L to 0.817 for VACVB5 (Fig. 2A and B, Supplementary Table S3, Supplementary Figure S3A). We then used the Youden Index thresholds to identify the antibody titre corresponding to optimal sensitivity and specificity to differentiate positive from negative samples. Of the eight antigens, VACVB5 at a titre above 0.082 IgGnu provided the best combined sensitivity and specificity (74% [95% CI: 59–82%] sensitivity and 81% [73–96%] specificity) to differentiate between Mpox true positive versus true negative samples. A chi-squared test was performed to assess the relationship between a history childhood smallpox vaccination status for those with available data and classification as “positive” (n = 15/187) or “negative” (8/63) based on this threshold. The analysis found no significant association (*p* = 0.39), indicating that childhood smallpox vaccination history did not influence the classification outcome. The sensitivity and specificity improved by restricting the ROC analysis to evaluate antibody responses post Mpox infection only. Using the 54 samples from the Mpox group as true positives and 78 samples from the Control group as true negatives respectively. The AUC ranged from 0.535 for D13L to 0.951 for B6R (Fig. 2C and D, Supplementary Table S4, Supplementary Figure S3B). The Youden Index provided a threshold for B6R titre above 0.075 IgGnu as the titre with the best sensitivity (89% [82–98%]) and specificity (94% [86–100%]) to differentiate between post infectious samples and Controls. Fisher's exact test showed a significant association (*p* = 0.04) between childhood smallpox vaccination history and classification as “positive” (n = 3/41) or “negative” (n = 0/79), suggesting a possible relationship between childhood smallpox vaccination and B6R titres post Mpox infection.

### Using the assay to differentiate between infection and vaccination induced immune responses

**Fig. 3:**
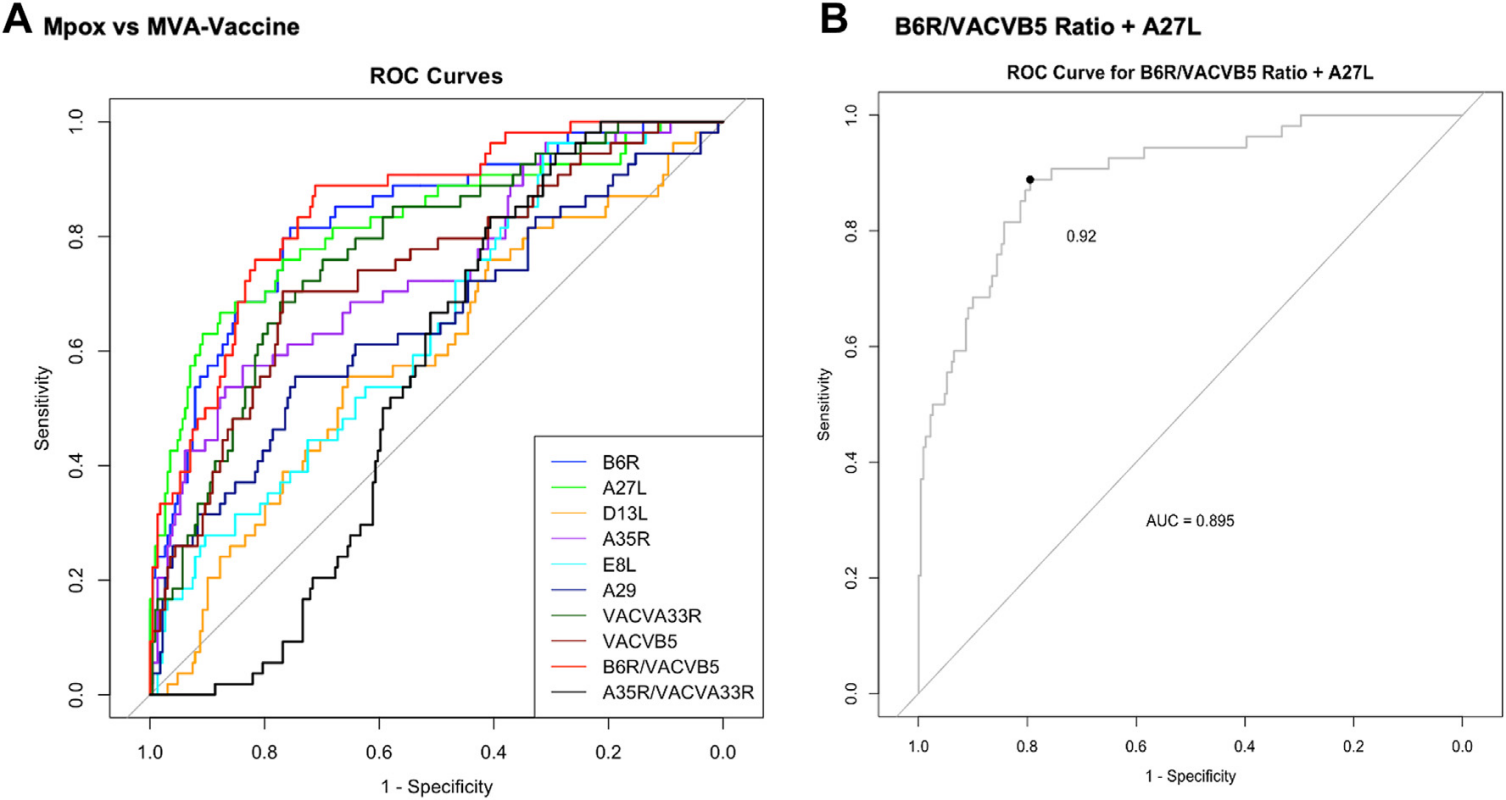
**Differentiation of infection and vaccination induced immunity**. **A:** ROC curve for all antigens and MPXV/VACV homologue ratios generated by comparing positive samples (Mpox, n = 54) to negative samples (MVA Vaccine, n = 229). **B:** ROC curve for B6R/VACVB5 ratio + A27L IgGnu generated by comparing positive samples (Mpox, n = 54) to negative samples (MVA Vaccine, n = 229).

To determine if the assay could differentiate between antibody responses arising from Mpox infection versus vaccination, ROC curves were generated comparing Mpox samples (n = 54) to MVA Vaccine samples (n = 229) for the eight antigens and the two MPXV/VACV homologue IgG titre ratios. The AUC ranged from 0.550 to 0.850 (Fig. 3A, Supplementary Table S5, Supplementary Figure S3C). The B6R/VACVB5 ratio performed best in differentiating post-infectious from post-vaccination immune responses (AUC 0.850), while the MPXV A27L antibody (targeting a region that is present in Mpox but is not present in the MVA-Vaccine),^27^ also performed well with an AUC of 0.822. Combining the A27L IgGnu with the B6R/VACVB5 ratio [sum of (B6R/VACVB5) + A7L IgGnu] resulted in the highest performing discriminator of infection from vaccination, with an AUC of 0.895. Using the Youden Index thresholds to identify the optimum threshold a [(B6R/VACVB5) + A27L IgGnu] value of 0.92 provided the best combined sensitivity (89% [80–96%]) and specificity (80% [74–84%]) (Fig. 3B) to differentiate post infection from post vaccination immune responses. Within the Mpox group, 8/54 (15%) samples were from participants with previous infection who had also received the MVA Vaccine; 7/8 (88%) of these samples were identified as infection using this threshold, highlighting its utility in differentiating infection even in those with a history of both infection and vaccination.

### Dynamic change in antibody responses post Mpox Infection and vaccination

We evaluated change in antibody responses over time using VACB5 IgG titres, as this marker had the best combined sensitivity and specificity to evaluate antibodies both post infection and vaccination.

**Fig. 4:**
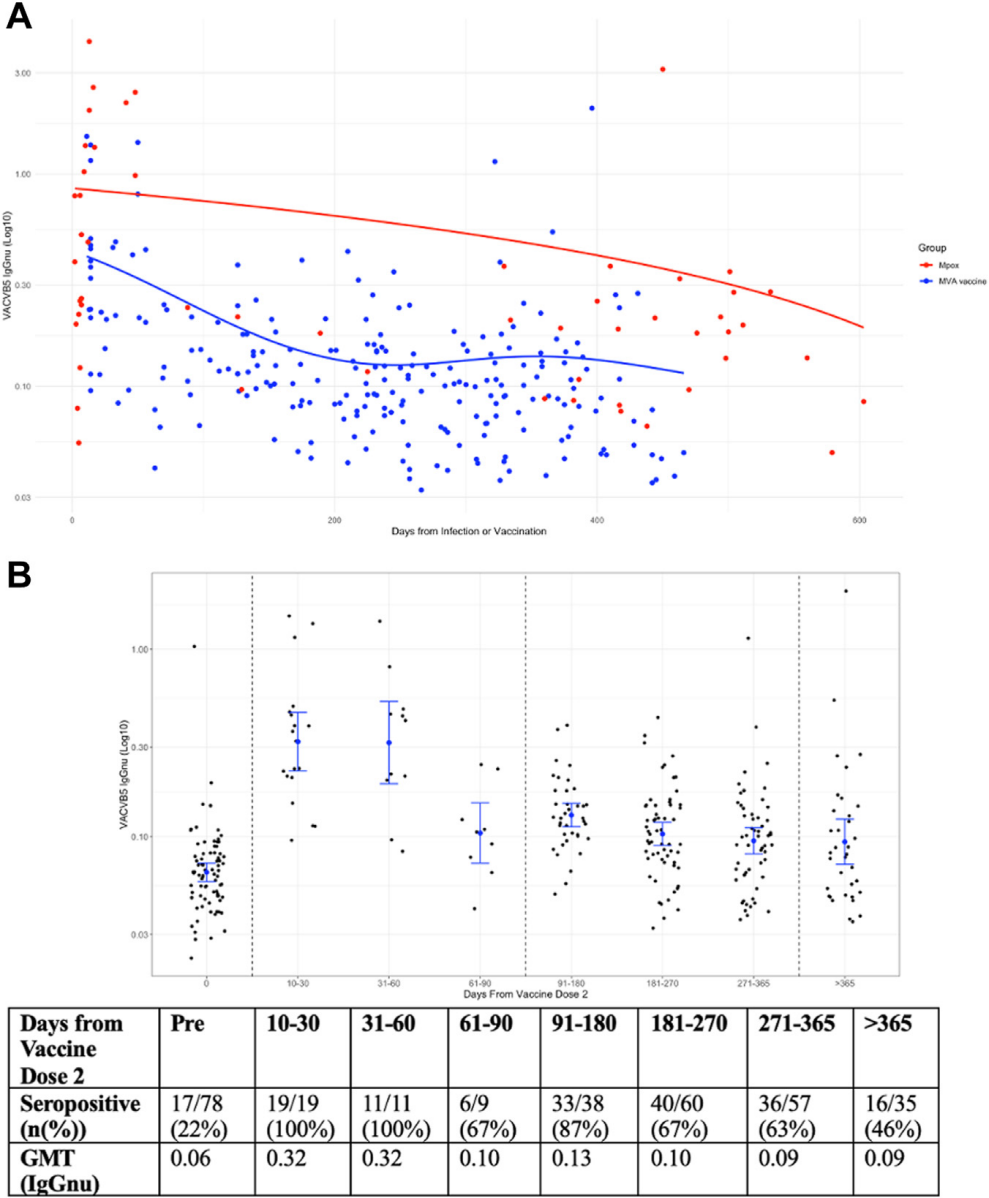
**Antibody responses over time**. **A:** VACV BS lgG Responses over time post MVA-Vaccination and Mpox Infection. **A:** Change in VACVB5 IgGnu titres over time since onset of symptoms of Mpox or dose 2 of MVA vaccination. We modelled change in VACVB5 IgGnu over time using scatter plots with superimposed curves fitted using generalised additive mixed models (GAMM), with a Gaussian link function and time since symptom onset or vaccination fitted as a spline. **B:** VACV BS IgG Responses over time post MVA-Vaccination. **B:** VACVB5 IgGnu titres over time before and after MVA vaccination. The centre of the error bar represents the geometric mean titre with the error bars representing the 95% confidence interval of the GMT. Seropositive is defined as the threshold identified by the Youden Index with the optimum sensitivity and specificity (0.082 IgGnu) from the ROC curve analysis for VACVB5 IgG.

In the Mpox group sampled at a median (IQR) of 277 (11–449) days from infection, VACVB5 IgG titres peaked shortly after onset of symptoms and declined over time since infection (Fig. 4A). However, for the duration of follow up, the aggregated mean titre (VACVB5 IgGnu) of 0.52 IgGnu remained detectible and above the previously defined threshold of 0.082 IgGnu. 20 (83.33%) samples evaluated after one year [median (IQR) 467 (417–502) days] were seropositive for VACVB5.

In the MVA Vaccine group, we evaluated VACV B5 IgG in 78 participants pre vaccination and in 229 samples taken at a median (IQR) of 238 (138–327) days post completion of MVA vaccination. The trajectory of antibody responses was not linear and similar to the Mpox group. VACV B5 IgG antibody responses increased rapidly following MVA vaccination, peaking at 11 days post completion of the vaccination course. However, in contrast to the Mpox group, post vaccine antibody titres rapidly declined until approximately 90 days post vaccination. Thereafter, VACB5 titres plateaued before a further declining, reaching lowest levels at 466 days post vaccine (Fig. 4A). Overall, the aggregated mean VACB5 titre of 0.18 IgGnu in the MVA vaccine group was significantly lower than the Mpox group (0.52 IgGnu, *p* < 0.0001). Using a threshold antibody VACB5 titre of 0.082 IgGnu, 36 (92.31%) samples within 90 days of vaccination were considered seropositive. In contrast to the Mpox group, this reduced to 109 (70.32%) samples from 90 days to one year to only 16 (45.71%) samples at one year [median (IQR) 399 (379–428) days] (Fig. 4B).

## Discussion

In response to the global Mpox outbreak, we have developed a quantitative, serologic assay incorporating three OPXV antigens (B6R, VACB5, and A27L) that when used together can accurately detect immune responses to both Mpox infection (B6R) and vaccination (VACB5) as well as differentiate between post infection and post vaccination immune responses [(B6R/VACB5) + A27L]. This assay provides a significant advancement in our ability to monitor and evaluate immune responses to an emerging infection.

A serological assay to assess immunity to Mpox is urgently needed to enhance our understanding of humoural immune responses to both infection and vaccination. This study initially quantified IgG responses to thirteen OPXV antigens, and identified three key antigens (MPXV B6R, A27L, and VACV B5) that provided best sensitivity and specificity to evaluate antibody responses to Mpox infection and vaccination. Multiplex IgG testing against these three antigens using a commonly available electrochemiluminescence (ECL) platform offers the potential for rapid transfer of this technology to low and middle-income countries (LMIC) such as the DRC, where Mpox cases are rising, and where serological assays will enable enhanced diagnosis and population-level surveillance.

Neutralising antibodies are used as a correlate of protection for many vaccine-preventable diseases. A high correlation between VACV IgG titres and MPXV 50% plaque reduction neutralisation test (PRNT_50_) titres was demonstrated in vaccinated individuals.^15^ However, unlike serological assays, neutralisation assays, particularly gold standard live virus neutralisation assays are labour intensive, expensive, and require both skilled staff and high biosafety levels. These requirements hinder their application in large-scale vaccine trials, routine clinical settings and in LMIC.

Measurement of the MPXVB6R IgG provided the best sensitivity and specificity to quantify post-infectious antibody responses while it's analogue VACVB5 IgG was optimal for quantification of vaccination induced immunity. VACVB5 antibodies are primarily responsible for the EEV neutralising capacity of human Vaccinia Immunoglobulin.^28^ Similarly, human neutralising antibodies targeting MPXVB6R demonstrate protection against VACV infection.^29^ Although further work is needed to define how well levels of binding VACVB5 or B6R IgG correlate with MPXV-specific viral neutralisation capacity, these data highlights the prominent role of both VACVB5 and MPXVB6R as potential correlates of immunity and supports the use of these antigens within a serological assay to quantify a clinically relevant IgG response to Mpox infection or vaccination.

Although challenging given the cross-reactivity within the OPXV genus, a serological assay capable of accurately differentiating between antibodies induced by infection and vaccination is critically important particularly given the potential for asymptomatic infection (and resulting immunity) as well as reports of breakthrough Mpox infections in previously vaccinated individuals.^23^ Previous studies have demonstrated that A27L IgG can differentiate between infection and vaccine-induced immunity with a sensitivity of 87.5% and a specificity of 96.8%.^23^ Through examination of a large number of potential antigenic targets, the current study enhances this approach by combining A27L with the B6R/VACVB5 ratio, thereby optimising the sensitivity and specificity of the test to differentiate infection from vaccine-induced immunity. Of note, this approach proved effective even when applied to a population with a history of both infection and vaccination, with 88% of such individuals correctly classified using the [(B6R/VACB5) + A27L] algorithm. Given the homology between MPXV clade I and II, this assay offers the potential to enhance global outbreak responses. Such a serological test, is pivotal for effective serosurveillance and could guide decisions on vaccination strategies, outbreak management, and public health interventions. It is crucial to further understand the epidemiology of Mpox, including transmission patterns and population immunity levels.

On a population level, VACVB5 IgG responses post vaccination were significantly lower than those post Mpox infection and waned considerably over time. Just 46% of those sampled over a year after vaccination had a seropositive VACVB5 IgG response, compared to 83% post infection. Although MVA vaccination has demonstrated non-inferior immunogenicity to the ACAM2000 vaccine in terms of peak serum neutralisation titres,^11^ little is known regarding the long-term immunogenicity of the MVA vaccination. The current study with its follow-up period of eight months post completion of MVA vaccination and nine months post Mpox infection provides valuable insights into the dynamics of IgG titres over time. Historically, live attenuated, vaccines such as those for measles and rubella, rather than non-replicating vaccines, have provided the most effective protection against viral infection and considered the prototype inducers of lifelong immunity.^30^ Using a modified non-replicating viral vector such as included in the MVA-vaccine results in abrogated immunogenicity compared to natural infection and may require booster doses to maintain effective circulating antibody titres. Although, lifelong immunity to Mpox post infection is presumed given the experience with smallpox, there have been reported cases of reinfections with Mpox.^23^ Waning antibody titres post infection in specific susceptible individuals, similar to what we observed in our study may be one factor that contributes to risk of re-infection, although further research is required to determine the relative role of waning antibody levels on risk of reinfection.

Limitations to this study include a limited demographic consisting of entirely adult participants assigned male sex at birth. This reflects the at-risk population from this specific outbreak. A previous meta-analysis demonstrated antibodies following MVA-vaccination were significantly higher in males than females.^31^ Females and children may also exhibit different patterns of antibody binding and our findings should be validated in a population of women and children. Although a large cohort study, this study was not designed to evaluate the impact of covariates such as age, HIV, and childhood smallpox vaccine on antibody responses. These factors could be examined in a structured clinical trial, such as MPOX-VAX (EUCT 2023-507881-19-00). All participants with Mpox had MPXV clade IIb disease acquired through sexual transmission. While similar antibody binding has been reported between individuals with Clade IIa and Clade IIb infection,^18^ and the antigenic targets chosen for this assay share homology across clades, further research is needed to evaluate the antibody response to MPXV clade Ib, currently responsible for the public health emergency in the DRC, where the majority of cases have been in women and children.^5^ Furthermore, the study population was predominantly composed of participants of caucasian race, with limited participants of African race as a notable limitation. Further studies are necessary to validate these findings within African populations to ensure generalisibility. Additionally, there is no international standard unit for binding antibody titres to OPXV antigens; therefore, normalisation of the IgG titres were based on the IgG titres of the plasma controls. For this assay to be replicated in multiple laboratories the development of an international standard unit for antibody titres is required alongside validation on other immunoassay platforms. Furthermore, the absence of neutralisation data limits our ability to determine the clinical relevance of the antibody thresholds as correlates of immunity. Correlation of these antibody titres with virus neutralisation assays, as demonstrated in other viral infections such as SARS-CoV-2,^32^ is essential to determine the predictive efficacy of vaccination induced antibody titres for protective immunity.

Despite these limitations, this study describes the development of anOPXV serological assay incorporating three key antigens (B6R, A27L, and VACVB5) that can effectively quantify and differentiate IgG responses to Mpox infection and vaccination and evaluated the antibody responses to infection and vaccination over time. Multiplex IgG testing against these three antigens could facilitate swift deployment of this technology to LMIC countries at the epicentre of the Mpox outbreak to enable enhanced diagnosis and population-level surveillance.

## Contributors

All authors have read and approved the final version of the manuscript.

JB: Conceptualisation, Formal analysis, Investigation, Methodology, Writing – original draft, Writing – review & editing. GS: Formal analysis, Investigation, Methodology, Writing – review & editing. AGL: Formal analysis, Investigation, Methodology, Writing – review & editing. DA: Methodology, Writing – review & editing. PD: Writing – review & editing. AL: Writing – review & editing. LBLN: Supervision, Writing – review & editing. COB: Supervision, Data curation, Writing – review & editing. SS: Data curation, Writing – review & editing. JAOH: Writing – review & editing. AC: Writing – review & editing. MH: Writing – review & editing. CK: Data curation, Writing – review & editing. CS: Writing – review & editing. EdB: Writing – review & editing. VG: Supervision, Writing – review & editing. PWGM: Conceptualisation, Formal analysis, Funding acquisition, Methodology, Resources, Writing – original draft, Writing – review & editing. ERF: Conceptualisation, Formal analysis, Funding acquisition, Methodology, Resources, Writing – original draft, Writing – review & editing.

## Data sharing statement

The data used for this analysis can be made available upon reasonable request to the corresponding author.

## Declaration of interests

J.B has received honoraria and/or travel grants from AstraZeneca, ViiV Healthcare and GSK.

P.W.G.M has received honoraria and/or travel grants from Janssen Cilag, Gilead Sciences, MSD, AstraZeneca, a member of advisory boards for AstraZeneca and ViiV Healthcare and has been awarded grants from Gilead Sciences and GlaxoSmithKline Ireland outside the submitted work. L.B.L.N has been awarded grants from Pfizer, Astrazeneca, Sanofi, Osivax, Linkyvax, MSD, GSK, Moderna, has received consulting fees from Pfizer, CEMKA, AstraZeneca, Gilead Sciences and has received honoraria and/or travel grants from Sanofi, Pfizer, Valneva, AstraZeneca and GSK. J.O.H has been awarded grants from Janssen Scientific. E.D.B has received honoraria from AstraZeneca. C.O.B has been awarded grants from Abbott. M.H is the director of education of ESCMID. All other authors declare no competing interests.
