# Supplementary material for: Development and validation of a quantitative Orthopoxvirus immunoassay to evaluate and differentiate serological responses to Mpox infection and vaccination
Source: eBioMedicine. 2025 Feb 22;113:105622. doi: 10.1016/j.ebiom.2025.105622 (PMC11904509; doi:10.1016/j.ebiom.2025.105622)
Supplement: Supplementary Figure S1 [file mmc1.docx]

**Supplementary Materials**

**Methods:**

**Electrochemiluminesce Assay**

Recombinant MPXV and VACV protein antigens were sourced as follows: A33R, A27L, H3L, A26L, A35R, D13L, (Abbexa, Cambridge, UK), and B6R, A29, M1R, E8L, VACV A27L, VACV A33R, VACV B5 (SinoBiological, Beijing, China). Each antigen was reconstituted in sterile water to a stock solution and stored at -80°C. 96 well high-bind plates (MSD, Rockville, MD) were washed 3 times with phosphate buffered saline and 0.05% tween (PBS tween) (Bio Sciences Ltd., Ireland). Each plate was coated separately with an antigen and the concentration of the coating antigen was determined using the maximum coating capacity of the high bind plates (5 picomoles of antigen per well) and incubated overnight at 4°C. Plates were washed 3 times with PBS tween and then blocked using 150ul of 1% blocker A (MSD) and incubated for 120 minutes at room temperature (RT). Thawed, ethylenediamine tetraacetic acid (EDTA)-derived plasma, diluted to 1:500 in MSD D100 were added (25ul of diluted plasma in duplicate per well). Seven biological controls were included on each plate; three plasma samples from participants with Mpox infection, three plasma samples from participants with previous MVA vaccination, and one negative plasma sample. Plates were then incubated for 90 minutes at RT, washed, following which MSD SULFO-TAG-labelled goat anti-human IgG secondary antibody (RRID:AB_2905663), diluted with MSD D100 was added at a concentration of 1μg/ml and the plate was further incubated for one hour at RT. Plates were washed 3 times with PBS tween and 150ul of MSD GOLD read buffer A containing ECL substrate was added. Plates were placed in a MESO QuickPlex SQ 120 instrument (MSD). Analysis was performed using MSD Discovery Workbench Software Version 4.0. Turnover time for the assay was 24 hours per plate. IgG titres were normalised and expressed as a ratio to the biological plate controls as IgG normalised units (IgGnu).

**Assay performance**

Data on intra- and inter-assay CV for each individual antigen is listed in Supplementary Table 6. The mean (95%CI) intra-assay coefficient of variation (CV) for 400 plasma samples run in duplicate on the same plate across all 13 antigens was 4.78 (4.67-4.89)%. The mean (95% CI) inter-assay CV, derived from 7 control samples run in duplicate across 10 plates across multiple days by multiple operators, was 15.81 (15.32-16.31)% for the 13 antigens tested.

**Supplementary Tables:**

**Table 1: MPXV and VACV antigens used in this assay, known functions and homology**

| **MPXV** | **Function*** | **VACV** |
| --- | --- | --- |
| **A29L** | IMV surface membrane fusion protein, binds cell surface heparan. | **VACV A27L**  (93.6% homology) |
| **A35R** | EEV envelope glycoprotein, needed for formation of actin-containing microvilli and cell-to-cell spread. | **VACV A33R**  **(**96.1% homology) |
| **B6R** | EEV envelope glycoprotein required for efficient cell spread, complement control protein-like. Major target of EEV-neutralising  antibodies. | **VACV B5**  (95.9% homology) |
| **A26L** | Unknown function. | Absent from VACV. |
| **A27L** | Unknown function in MPXV. VACV homologue function is as an IMV fusion suppressor. | Absent from MVA-BN. |
| **A33R** | Unknown function. |  |
| **D13L** | Unknown function. |  |
| **E8L** | IMV surface membrane protein, binds cell surface chondroitin sulfate, IMV adsorption to cell surface. |  |
| **H3L** | IMV heparan-binding surface membrane protein, attaches on the cell surface by binding to glycosaminoglycan (GAGs). |  |
| **M1R** | IMV surface membrane protein, mediates virus entry into cells  independently of GAGs. |  |

**Abbreviations:** IMV, Intracellular mature virions, EEV, Extracellular enveloped virions, MPXV, Monkeypox virus, VACV, Vaccinia virus

MPXV antigens based on clade I MPXV reference genome Zaire-96-I-169 (NCBI:txid619591). VACV antigens based on the VACV Copenhagen strain genome (NCBI:txid10249).

Homology between MPXV and VACV antigens calculated using the NCBI BLASTp tool.(8)

Known function of MPXV and VACV antigens summarised.(31-33)

**Table 2 Differences in IgG titres per group by antigens**

| **Antigen** | **Mpox**  **(n=54)**  **(GMT)** | **MVA-Vaccine**  **(n=229)**  **(GMT)** | **Control**  **(n=78**)  **(GMT)** | **Childhood Vaccine**  **(n=22)**  **(GMT)** | **Mpox vs Control**  ***p* value*** | **MVA-Vaccine vs Control**  ***p* value*** | **Mpox vs MVA-Vaccine**  ***p* value*** | **Control vs**  **Childhood Vaccine**  ***p* value*** |
| --- | --- | --- | --- | --- | --- | --- | --- | --- |
| **A27L** | 0.30 | 0.10 | 0.10 | 0.11 | <0.0001 | ns | <0.0001 | ns |
| **D13L** | 0.92 | 0.83 | 0.87 | 1.16 | ns | ns | 0.02 | 0.01 |
| **A35R** | 1.56 | 0.77 | 0.95 | 1.09 | <0.0001 | ns | <0.0001 | 0.05 |
| **E8L** | 0.60 | 0.44 | 0.38 | 0.36 | <0.0001 | 0.04 | 0.01 | ns |
| **B6R** | 0.26 | 0.08 | 0.05 | 0.05 | <0.0001 | <0.0001 | <0.0001 | ns |
| **A29** | 0.59 | 0.43 | 0.39 | 0.33 | <0.0001 | 0.02 | 0.01 | 0.02 |
| **VACVA33R** | 1.01 | 0.5 | 0.33 | 0.43 | <0.0001 | <0.0001 | <0.0001 | 0.01 |
| **VACVB5** | 0.28 | 0.12 | 0.06 | 0.06 | <0.0001 | <0.0001 | <0.0001 | ns |

**Abbreviations:** GMT, Geometric Mean Titre

**p* value calculated by Post-Hoc Dunn’s test following Kruskal-Wallis test.

**Table 3: Receiver Operating Characteristic Curves Analysis for Each Antigen (Mpox Infection and MVA Vaccination vs Negative)**

|  | **Threshold**  **(IgGnu)** | **AUC**  **(95% CI)** | **Sensitivity**  **(95% CI)** | **Specificity**  **(95% CI)** |
| --- | --- | --- | --- | --- |
| **A27L** | 0.142 | 0.567 (0.498- 0.636) | 0.357 (0.201- 0.689) | 0.795 (0.462- 0.936) |
| **D13L** | 0.934 | 0.528 (0.452-0.603) | 0.604 (0.296- 0.929) | 0.513 (0.154- 0.783) |
| **A35R** | 1.224 | 0.560 (0.497- 0.622) | 0.403 (0.226-0.633) | 0.821 (0.603- 0.974) |
| **E8L** | 0.332 | 0.594 (0.523- 0.665) | 0.753 (0.152- 0.827) | 0.423 (0.346- 0.974) |
| **B6R** | 0.065 | 0.789 (0.739- 0.839) | 0.650 (0.569- 0.781) | 0.885 (0.744- 0.962) |
| **A29** | 0.340 | 0.608 (0.537- 0.678) | 0.770 (0.258- 0.816) | 0.423 (0.372- 0.923) |
| **VACVA33R** | 0.382 | 0.780 (0.728- 0.832) | 0.721 (0.548- 0.816) | 0.756 (0.654-0.923) |
| **VACVB5** | 0.082 | 0.817 (0.767- 0.866) | 0.742 (0.590- 0.820) | 0.808 (0.731- 0.962) |

Abbreviations: AUC, Area under the curve, IgGnu, Immunoglobulin G normalised units

ROC curves were generated by comparing positive samples (Mpox group and MVA Vaccine group, n=283) to negative samples (Control group, n=78). Threshold IgGnu values were selected based on the optimum cut-off for sensitivity and specificity as selected by the Youden Index.

**Table 4: Receiver Operating Characteristic Curves (ROC) Analysis for Each Antigen (Mpox Infection vs Negative)**

|  | **Threshold**  **(IgGnu)** | **AUC**  **(95% CI)** | **Sensitivity**  **(95% CI)** | **Specificity**  **(95% CI)** |
| --- | --- | --- | --- | --- |
| **A27L** | 0.149 | 0.819 (0.742- 0.896) | 0.759 (0.648- 0.870) | 0.808 (0.718- 0.885) |
| **D13L** | 0.686 | 0.535 (0.435- 0.635) | 0.815 (0.704- 0.907) | 0.346 (0.244- 0.462) |
| **A35R** | 1.461 | 0.764 (0.675- 0.852) | 0.574 (0.444- 0.704) | 0.949 (0.897- 0.987) |
| **E8L** | 0.333 | 0.708 (0.620- 0.796) | 0.963 (0.907- 1.000) | 0.423 (0.321- 0.538) |
| **B6R** | 0.075 | 0.951 (0.912- 0.990) | 0.889 (0.815-0.981) | 0.936 (0.859- 1.000) |
| **A29** | 0.534 | 0.711 (0.620-0.802) | 0.556 (0.426- 0.685) | 0.833 (0.744- 0.910) |
| **VACVA33R** | 0.506 | 0.937 (0.896-0.978) | 0.852 (0.759- 0.944) | 0.897 (0.821- 0.962) |
| **VACVB5** | 0.115 | 0.922 (0.874-0.970) | 0.778 (0.667- 0.889) | 0.949 (0.897- 0.987) |

Abbreviations: AUC, Area under the curve, IgGnu, Immunoglobulin G normalised units

ROC curves were generated by comparing positive samples restricted to just infection samples (Mpox group, n=54) to negative samples (Control group, n=78). Threshold IgGnu values were selected based on the optimum cut-off for sensitivity and specificity as selected by the Youden Index.

**Table 5: Receiver Operating Characteristic Curves (ROC) Analysis for Each Antigen and MPXV/VACV Homologue (Mpox Infection vs MVA Vaccination)**

|  | **Threshold** | **AUC** | **Sensitivity**  **(95% CI)** | **Specificity**  **(95% CI)** |
| --- | --- | --- | --- | --- |
| **A27L (IgGnu)** | 0.180 | 0.822 (0.752 - 0.892) | 0.667 (0.537- 0.796) | 0.878(0.834- 0.917) |
| **D13L (IgGnu)** | 0.946 | 0.589 (0.504-0.675) | 0.556 (0.426- 0.685) | 0.656 (0.598- 0.716) |
| **A35R (IgGnu)** | 1.463 | 0.739 (0.661- 0.816) | 0.574 (0.444- 0.704) | 0.838 (0.790- 0.886) |
| **E8L (IgGnu)** | 0.334 | 0.640 (0.561- 0.718) | 0.963 (0.907- 1.000) | 0.306 (0.245- 0.367) |
| **B6R (IgGnu)** | 0.104 | 0.831 (0.768- 0.893) | 0.815 (0.648- 0.926) | 0.756 (0.672- 0.895) |
| **A29 (IgGnu)** | 0.538 | 0.650 (0.561-0.738) | 0.556 (0.426- 0.685) | 0.747 (0.690- 0.799) |
| **VACVA33R (IgGnu)** | 0.711 | 0.774 (0.707-0.841) | 0.685 (0.556- 0.796) | 0.773 (0.721- 0.825) |
| **VACVB5 (IgGnu)** | 0.177 | 0.739 (0.662-0.815) | 0.704 (0.574- 0.815) | 0.769 (0.712- 0.830) |
| **B6R/VACVB5 (ratio)** | 0.752 | 0.850 (0.795-0.904) | 0.889 (0.796- 0.963) | 0.712 (0.655- 0.769) |
| **A35R/VACVA33R (ratio)** | 2.200 | 0.550 (0.483- 0.617) | 0.833 (0.722- 0.926) | 0.406 (0.345- 0.467) |
| **B6R/VACVB5 (ratio) + A27L (IgGnu)** | 0.920 | 0.895 (0.847- 0.943) | 0.889 (0.796- 0.963) | 0.795 (0.742- 0.843) |
| **B6R + A27L (IgGnu)** | 0.333 | 0.859 (0.798- 0.921) | 0.778 (0.666- 0.889) | 0.873 (0.830- 0.913) |

Abbreviations: AUC, Area under the curve, IgGnu, Immunoglobulin G normalised units

ROC curves were generated by comparing positive samples (Mpox group, n=54) to negative samples (MVA-Vaccine group, n=229). Threshold IgGnu values were selected based on the optimum cut-off for sensitivity and specificity as selected by the Youden Index.

**Table 6 – Intra- and Inter-Assay Variability**

| **Antigen** | **Intra-Assay CV**  **(Mean (95% CI (%))** | **Inter-Assay CV**  **(Mean (95% CI (%))** |
| --- | --- | --- |
| All antigens | 4.78 (4.67-4.89) | 15.81 (15.32-16.31) |
| A27L | 4.77 (4.74-4.80) | 13.21 (11.26-15.16) |
| A35R | 4.77 (4.73-4.81) | 24.27 (19.81-28.73) |
| A33R | 6.04 (6.01-6.08) | 18.77 (16.54-21.00) |
| D13L | 4.31 (4.29-4.33) | 14.36 (12.91-15.80) |
| H3L | 4.09 (4.06-4.11) | 17.91 (15.61-20.22) |
| A26L | 4.01 (3.98-4.04) | 13.92 (11.99-15.85) |
| E8L | 4.11 (4.09-4.14) | 14.63 (12.69-16.57) |
| B6R | 4.83 (4.80-4.86) | 15.11 (12.74-17.48) |
| M1R | 4.57 (4.54-4.60) | 14.34 (12.60-16.09) |
| A29 | 5.17 (5.13-5.21) | 14.63 (11.96-17.30) |
| VACVA27L | 5.90 (5.85-5.94) | 14.58 (13.22-15.95) |
| VACVA33R | 5.16 (5.13-5.20) | 16.89 (13.84-19.94) |
| VACVB5 | 4.41 (4.38-4.43) | 12.94 (11.46-14.42) |

Intra-assay CV calculated from 383 plasma samples run in duplicate on the same plate for each antigen.

Inter-assay CV derived from 7 control samples run in duplicate across 10 plates across multiple days by multiple operators for each antigen

Abbreviations: CV: Coefficient of Variation

**Supplementary Material Figures**

**
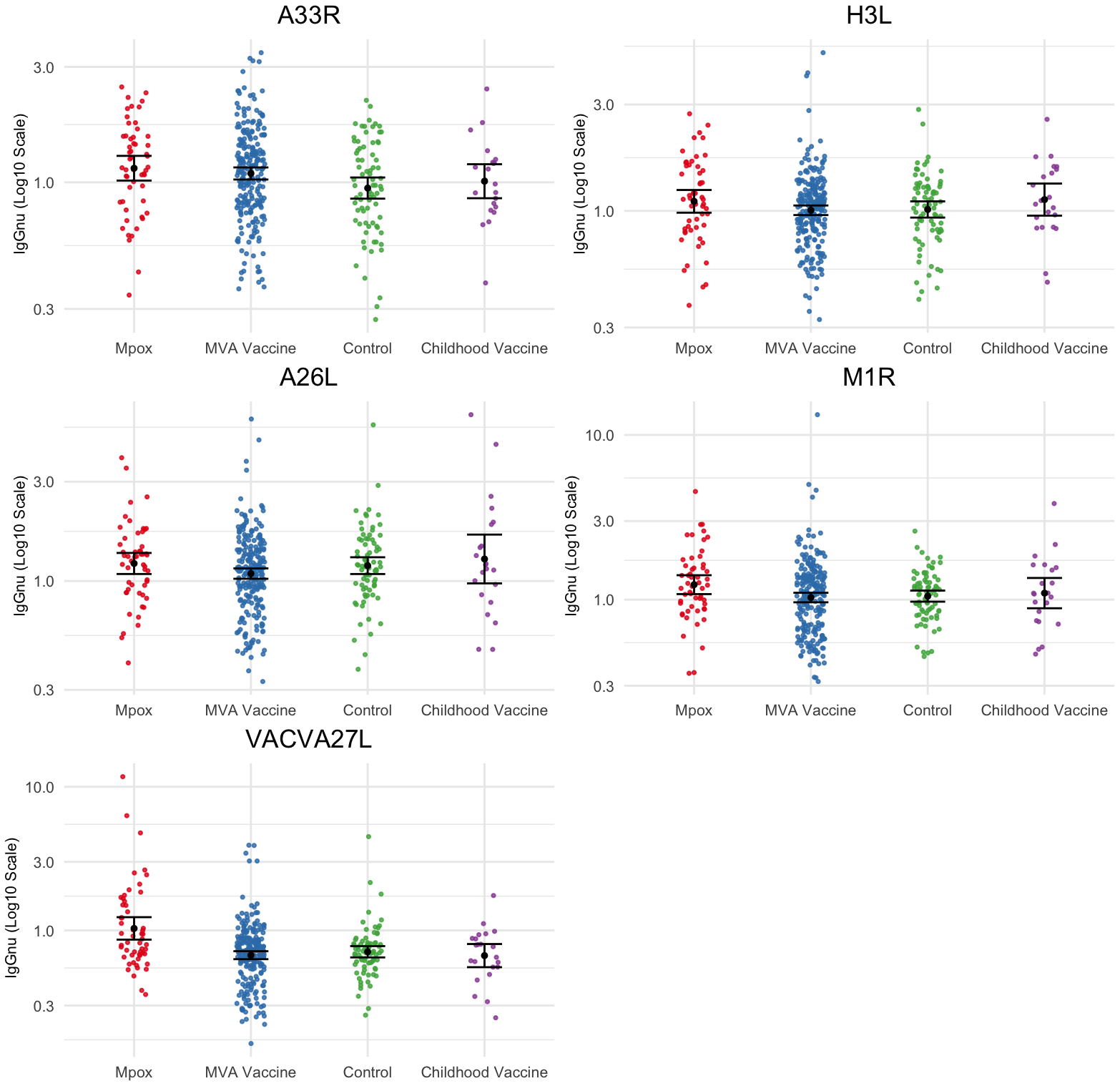
Figure 1: Antigens with no significant differences in IgG titres across groups**

Each graph represents the IgG titres for that antigen for each group. Mpox (n=54), MVA Vaccine (n=229), Control (n=78), Childhood Vaccine (n=22). The error bars represent the geometric mean titre (GMT) and the 95% confidence interval of the GMT for each group. Each antigen in this figure did not have significantly different titres across the four groups by Kruskal-Wallis Test (all *p*>0.05*).*

**Figure 2: Ratio of IgG Titres between MPXV and VACV homologues**

**
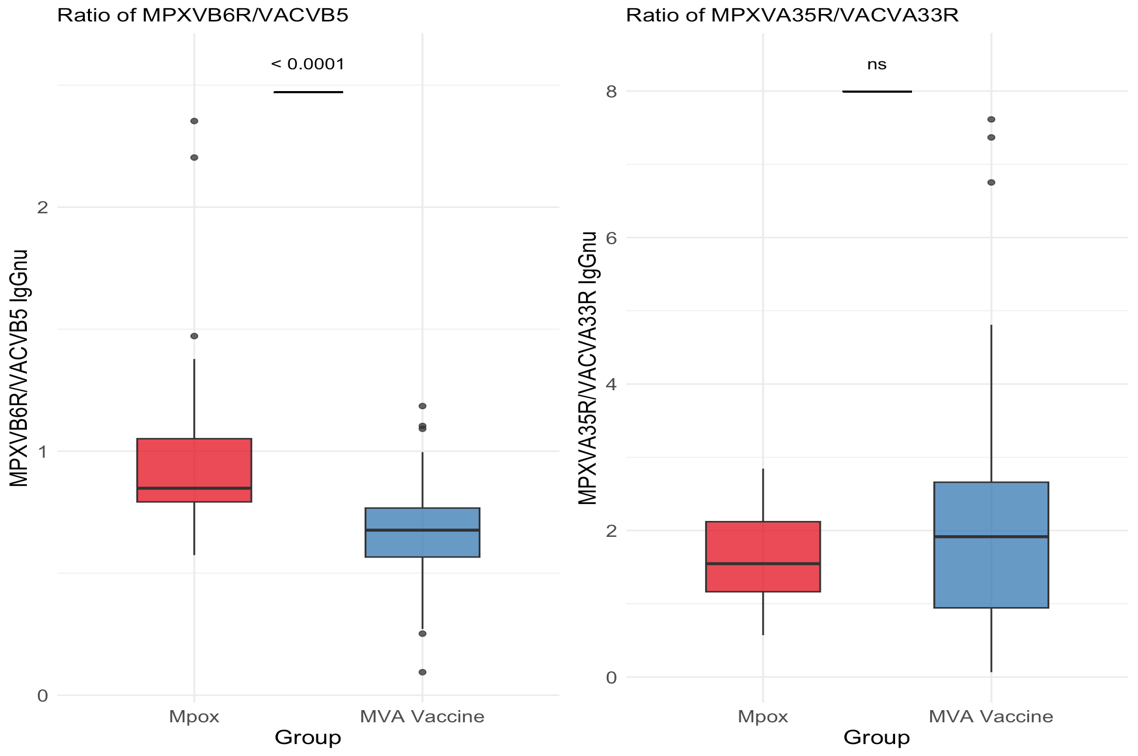
**

Boxplots comparing the ratio of MPXV IgG titres and respective VACV IgG homologues titres. MPXV B6R/VACVB5 IgG Ratio and MPXV A35R/VACVA33R in Mpox group (n=54) and MVA Vaccine group (n=229) The centre of the boxplots represents the median, the bounds of the box represent the interquartile range, and the whiskers represent 1.5 times the interquartile range with dots representing the outliers.

*P* values shown represent between group differences as per Mann-Whitney U test.

Abbreviations: ns, not significant

**
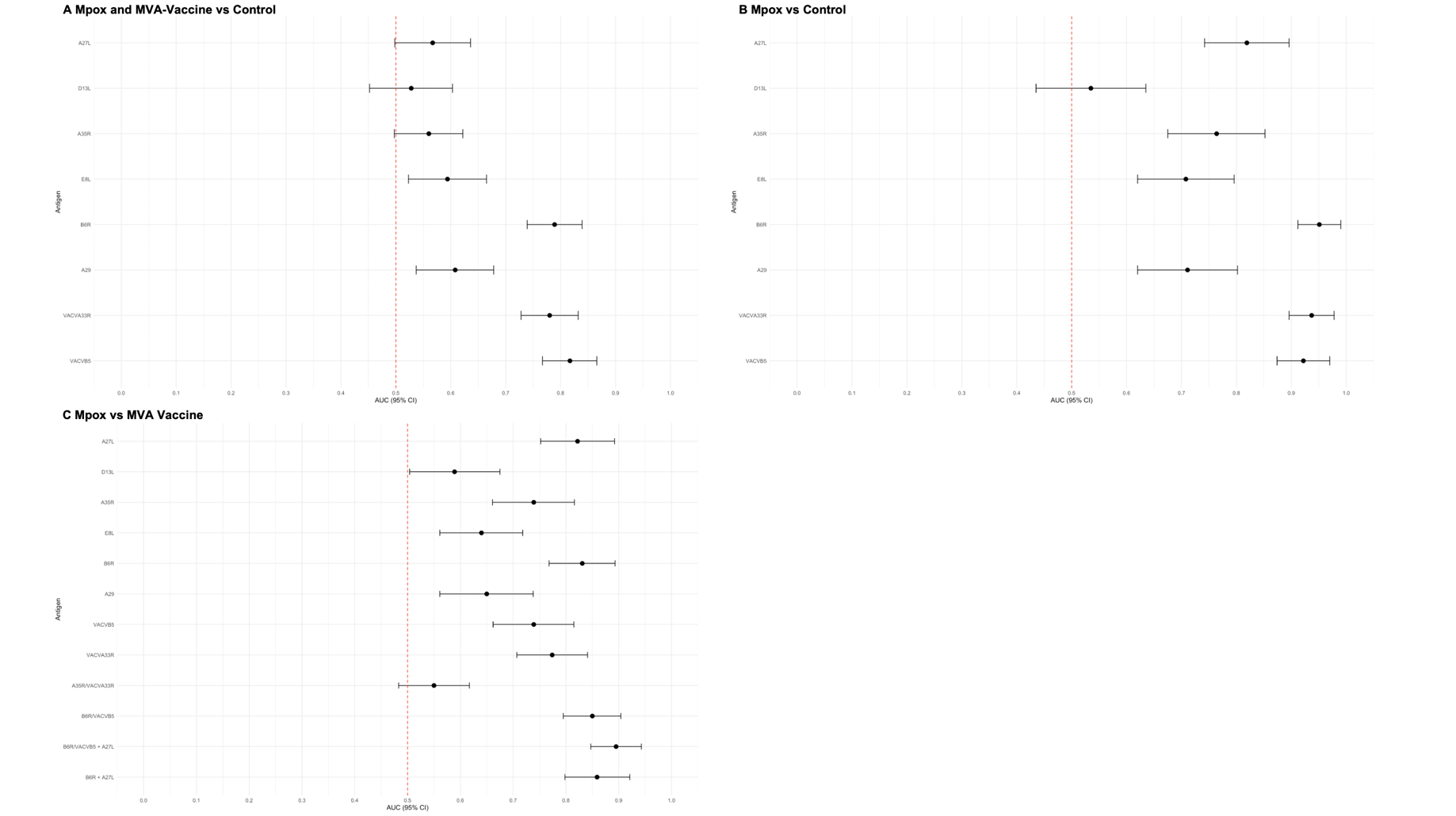
Figure 3: Forest plot of AUC and 95% CI for assay performance**

**A:** AUC (95% CI) for ROC curve for all antigens generated by comparing positive samples (Mpox and MVA Vaccine group, n=283) to negative samples (Control group, n=78).

**B:** AUC (95% CI) for ROC curve for all antigens generated by comparing positive samples restricted to infection only (Mpox group, n=54) to negative samples (Control group, n=78).

**C:** AUC (95% CI) for ROC curve for all antigens and MPXV/VACV homologue ratios generated by comparing positive samples (Mpox, n=54) to negative samples (MVA Vaccine, n=229).

Abbreviations: AUC, Area under the curve, 95%CI (95% Confidence Interval), IgGnu, Immunoglobulin G normalised units
